# Sézary Syndrome with CD4/CD8 Double-Negative Neoplastic T Cells in Peripheral Blood

**DOI:** 10.1155/2021/5527725

**Published:** 2021-06-01

**Authors:** Yuki Kageyama, Kenshiro Tsuda, Yuma Nato, Keiki Nagaharu, Kazutaka Suzuki, Akihiko Sawaki, Hiroyuki Miyashita

**Affiliations:** ^1^Department of Hematology, Yokkaichi Municipal Hospital, 2-2-37 Shibata, Yokkaichi 510-8567, Japan; ^2^Department of Hematology and Oncology, Mie University Graduate School of Medicine, 2-174 Edobashi, Tsu 514-8507, Japan; ^3^Department of Dermatology, Yokkaichi Municipal Hospital, 2-2-37 Shibata, Yokkaichi 510-8567, Japan

## Abstract

Sézary syndrome is a rare leukemic type of cutaneous T-cell lymphoma characterized by the presence of neoplastic T cells with cerebriform nuclei (Sézary cells) in the skin, lymph nodes, and peripheral blood. Typical Sézary cells have a CD3^+^CD4^+^CD8^–^ phenotype; however, in cases of the aberrant loss of antigens on Sézary cells, especially the loss of critically important T-cell antigens such as CD4, there is a possibility of misdiagnosing the disease or underestimating the tumor burden of the disease. Here, we report a rare case of Sézary syndrome with CD4/CD8 double-negative Sézary cells in the peripheral blood. Most of the Sézary cells in the peripheral blood had lost CD4 expression, and we diagnosed the disease and evaluated the tumor burden by multicolor flow cytometry. Intriguingly, the Sézary cells showed a typical CD4^+^CD8^–^CD7^–^ phenotype in the skin even though the cells in the peripheral blood lacked CD4. The patient responded well to treatment with bexarotene and narrow-band ultraviolet B therapy. Analysis by multicolor flow cytometry is essential to diagnose this rare type of Sézary syndrome and evaluate the tumor burden.

## 1. Introduction

Sézary syndrome (SS) is a rare leukemic type of cutaneous T-cell lymphoma defined by erythroderma, generalized lymphadenopathy, and the presence of neoplastic T cells with cerebriform nuclei (Sézary cells) in the skin, lymph nodes, and peripheral blood (PB) [1]. In addition to demonstrating clonally related neoplastic T cells in the skin and PB, one or more of the following criteria are required to diagnose SS: an absolute Sézary cell count ≥1,000/*µ*L; an expanded CD4^+^ T-cell population resulting in a CD4 : CD8 ratio of ≥10; or the loss of one or more T-cell antigens. Because of the morphologic heterogeneity of Sézary cells, flow cytometric analysis of PB is essential for diagnostic purposes and tumor burden monitoring during follow-up [2, 3]. Typical Sézary cells have a CD3^+^CD4^+^CD8^–^ phenotype and characteristically lack CD7 and CD26 [4]. In cases with an aberrant loss of CD4 on Sézary cells, it is difficult to diagnose the disease and to evaluate the tumor burden using routine flow cytometric analysis. In this article, we report a rare case of SS with CD4^–^CD8^–^ Sézary cells in PB detected by multicolor flow cytometry.

## 2. Case Presentation

**Figure 1:**
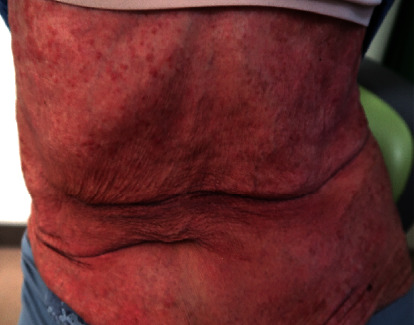
Image of the patient's chest and abdomen with erythroderma.

**Figure 2:**
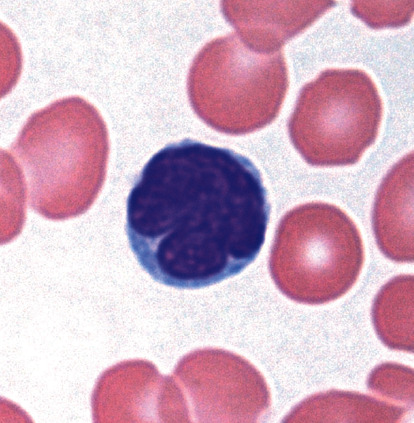
May–Giemsa staining of a blood smear showing abnormal cells with cerebriform nuclei (×1,000).

**Figure 3:**
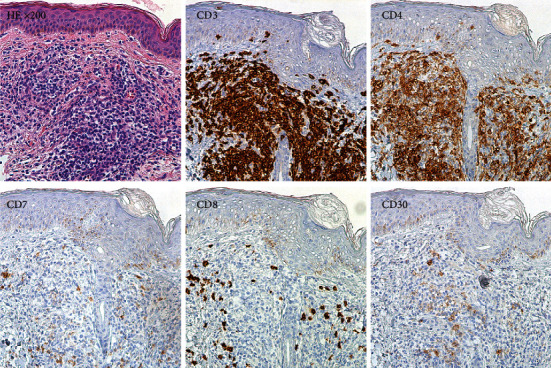
Hematoxylin-eosin (HE) staining and immunohistochemical staining of skin biopsy specimens (×200). HE staining shows the infiltration of abnormal lymphocytes in the superficial dermis (upper left). Immunohistochemical staining shows abnormal lymphocytes are positive for CD3 (upper middle) and CD4 (upper right), but negative for CD7 (lower left), CD8 (lower middle), and CD30 (lower right).

An 82-year-old woman presented to our hospital because of erythroderma with scattered invasive erythemas, nodules, and tumors on the face, trunk, and extremities (Figure 1). PB analysis revealed a white blood cell count of 14,730/*µ*L (reference range, 3.3–8.6 × 10^3^/*µ*L), with 28% abnormal cells containing cerebriform nuclei (Figure 2), a hemoglobin level of 12.2 g/dL (11.6–14.8 g/dL), and a platelet count of 311 × 10^3^/*µ*L (158–348 × 10^3^/*µ*L). The blood levels of lactate dehydrogenase and soluble interleukin-2 receptor were 362 IU/L (124–224 IU/L) and 2,139 U/mL (122–496 U/mL), respectively. A serologic test for human T-cell leukemia virus type 1 was negative. Computed tomography revealed no lymphadenopathy or hepatosplenomegaly. Skin biopsy specimens from a tumor on the left arm showed the infiltration of abnormal lymphocytes with medium- to large-sized nuclei containing convolutions in the superficial dermis (Figure 3). Immunohistochemical staining of skin biopsy specimens revealed abnormal lymphocytes expressing CD3 and CD4, but not CD7, CD8, CD20, or CD30. Despite approximately 30% of leukocytes in PB being morphologically abnormal, a small percentage of PB cells expressed CD4, and the CD4 : CD8 ratio analyzed by conventional two-color flow cytometry was 1.7.

**Figure 4:**
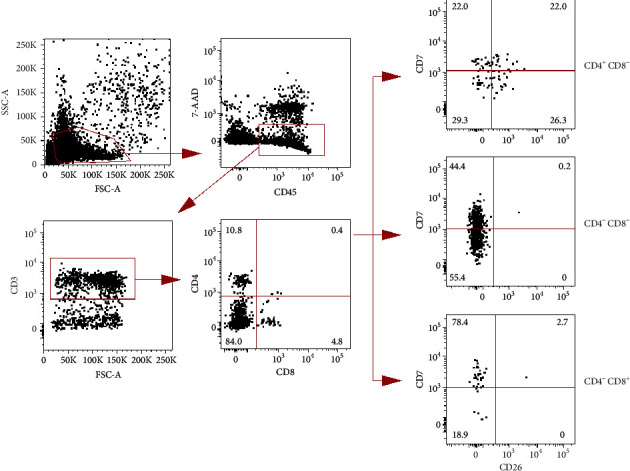
Results of multicolor flow cytometry of PB cells. After discriminating for dead cells and CD45^–^ cells, the remaining cells were gated on CD3^+^, and then three T-lymphocyte fractions, CD4^+^CD8^–^, CD4^–^CD8^–^, and CD4^–^CD8^+^, were analyzed for CD7 and CD26 expression. Percentages of each cell fraction are indicated in the plot areas. Abbreviations: FSC-A, forward scatter area; SSC-A, side scatter area; 7-AAD, 7-aminoactinomycin D.

**Figure 5:**
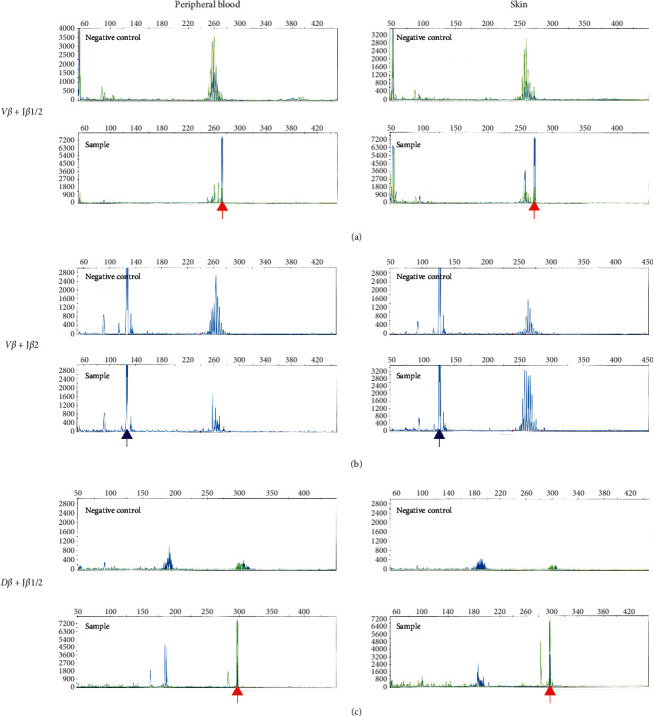
Results of TCR *β*-chain gene clonality assays. PCR products were produced and analyzed in accordance with the BIOMED-2 guidelines. The results of capillary electrophoresis of PCR products derived from PB (left) and skin (right) samples are shown. (a), (b), and (c) indicate the data of *Vβ *+* Jβ*1/2, *Vβ *+* Jβ*2, and *Dβ *+* Jβ*1/2, respectively. In each figure, an upper figure shows data from negative control and a lower shows data from sample. The vertical line shows fluorescence intensity, the horizontal line shows fragment size, red arrows show clonal peak, and blue arrows show non-specific peak.

Next, we performed a detailed phenotypical analysis by multicolor flow cytometry using a BD LSRFortessa™ cell analyzer (BD Biosciences, Franklin Lakes, NJ, USA). Among CD3^+^ T cells, 84.0% were CD4^–^CD8^–^, 10.8% were CD4^+^CD8^–^, and 4.8% were CD4^–^CD8^+^ (Figure 4). CD4^+^CD8^–^ T cells expressed CD7 and/or CD26 similar to normal helper T cells, whereas CD4^–^CD8^–^ T cells had a diminished expression of CD7 and lacked CD26 expression, consistent with the Sézary cell phenotype. T-cell receptor (TCR) *β*-chain gene clonality assays were performed in the PB and skin samples in accordance with the BIOMED-2 guidelines of polymerase chain reaction- (PCR-) based immunoglobulin/TCR clonality testing (performed by SRL, Inc., Tokyo, Japan) [5]. Both PB and skin samples showed a clonal peak in *Vβ *+* Jβ*1/2 and *Dβ *+* Jβ*1/2, and the size of those PCR products was completely matched between both specimens as 273 bp in *Vβ *+* Jβ*1/2 and 296 bp in *Dβ *+* Jβ*1/2 (Figure 5). The results suggested that tumor cells in PB and skin were clonally identical, consistent with the pathological definition of SS.

Based on these clinical, histopathological, immunohistochemical, and genetic features, this case was diagnosed as stage IVA1 (T4N0M0B2) SS according to the International Society for Cutaneous Lymphoma/European Organization for Research and Treatment of Cancer system. The patient was treated with bexarotene and narrow-band ultraviolet B therapy, which effectively reduced the cutaneous lesions and PB involvement. The best global response of the disease was a partial response based on the proposed response criteria [6]. The patient has been under this treatment for more than 9 months.

## 3. Discussion

In SS, the aberrant loss of T-cell antigens is frequently observed. The loss of CD7 and/or CD26 on neoplastic T cells is sensitive and highly specific for SS [7]. The slightly dim expression of CD4 on Sézary cells was reported in a case series [3]; however, CD4^−^ Sézary cells have rarely been described. A previous study reported a case of SS where 66% of the CD3^+^ fraction was CD4^–^CD8^–^ Sézary cells [3]. Unlike our case, the Sézary cells expressed CD7 and partially lacked CD26. Because the criteria for blood involvement including an absolute Sézary cell count ≥1,000/*µ*L and a CD4 : CD8 ratio of ≥10 are greatly affected in cases where CD4 molecules are lost in the PB, it is difficult to diagnose SS using conventional flow cytometry. In such cases, the analysis of Sézary cells using multicolor flow cytometry is essential. Intriguingly, our case showed typical CD4^+^CD8^–^CD7^–^ neoplastic cell invasion of the skin even though Sézary cells in PB lacked CD4. In addition, the results of PCR-based TCR *β*-chain gene rearrangement analyses suggested that tumor cells in skin and PB were clonally identical. Based on these findings, we were able to make a reliable diagnosis.

SS is an aggressive disease, with a 5-year overall survival rate of less than 40% [1,8]. Combination therapies with skin-directed and systemic therapies have been suggested as treatment regimens. We selected narrow-band ultraviolet B therapy as a skin-directed therapy and bexarotene, an oral retinoid, as systemic therapy. Although the patient responded well to the treatments, the potential relationship between the CD4^–^CD8^–^ phenotype and a good response to the treatment is unclear because of the scarcity of the disease. In mycosis fungoides, another subtype of cutaneous T-cell lymphoma, it was reported that patients with CD4^–^CD8^–^ tumor cells had an indolent clinical course typical of patients harboring CD4^+^ tumor cells [9].

In conclusion, CD4^–^CD8^–^ SS is a rare immunophenotype of SS. Analysis by multicolor flow cytometry was essential to diagnose the disease and evaluate the tumor burden. Further studies are needed to clarify the pathogenesis and prognosis of CD4^–^CD8^–^ SS.
